# Protein-coding genes in B chromosomes of the grasshopper *Eyprepocnemis plorans*

**DOI:** 10.1038/srep45200

**Published:** 2017-04-03

**Authors:** Beatriz Navarro-Domínguez, Francisco J. Ruiz-Ruano, Josefa Cabrero, José María Corral, María Dolores López-León, Timothy F. Sharbel, Juan Pedro M. Camacho

**Affiliations:** 1 Departamento de Genética, Facultad de Ciencias, Universidad de Granada, 18071 Granada, Spain; 2 Leibniz Institute for Plant Genetics and Crop Plant Research (IPK), D-06466 Gatersleben, Germany; 3 Department of Bioanalytics, Coburg University of Applied Sciences and Arts, Coburg, Germany; 4 Global Institute for Food Security, 110 Gymnasium Place, University of Saskatchewan, Saskatoon, Saskatchewan, S7N 4J8, Canada

## Abstract

For many years, parasitic B chromosomes have been considered genetically inert elements. Here we show the presence of ten protein-coding genes in the B chromosome of the grasshopper *Eyprepocnemis plorans*. Four of these genes (*CIP2A, GTPB6, KIF20A*, and *MTG1*) were complete in the B chromosome whereas the six remaining (*CKAP2, CAP-G, HYI, MYCB2, SLIT* and *TOP2A*) were truncated. Five of these genes (*CIP2A, CKAP2, CAP-G, KIF20A*, and *MYCB2*) were significantly up-regulated in B-carrying individuals, as expected if they were actively transcribed from the B chromosome. This conclusion is supported by three truncated genes (*CKAP2, CAP-G* and *MYCB2*) which showed up-regulation only in the regions being present in the B chromosome. Our results indicate that B chromosomes are not so silenced as was hitherto believed. Interestingly, the five active genes in the B chromosome code for functions related with cell division, which is the main arena where B chromosome destiny is played. This suggests that B chromosome evolutionary success can lie on its gene content.

Many supernumerary (B) chromosomes are rich in repetitive satellite and ribosomal DNA sequences, and their unusual meiotic dynamics and dispensable nature makes them an easy target for the integration and expansion of transposable elements (TEs)^1^,^2^. Many other kinds of DNA sequences could be interspersed among these repeats. This was first shown in the B chromosomes of rye (*Secale cereale*), which bear specific repeats and insertions of organellar DNA as well as gene-derived sequences showing fragmentation and pseudogenization^3^, including at least one functional gene^4^.

Although the high enrichment of repetitive DNA in B chromosomes hindered the detection of protein-coding genes in them, things have changed in the last decade by the discovery of proto-oncogenes and tumor-suppressor genes in the B chromosomes of several canid species^5^,^6^, H3 and H4 histone genes in those of the migratory locust^7^, and other protein-coding genes in the B chromosomes of a cichlid fish^8^ and two cervid species^9^,^10^. In addition, Valente *et al*. analyzed the gene content of B chromosomes by comparing Illumina sequences from 0B and 2B genomes in the cichlid fish *Astatotilapia latifasciata*, with subsequent confirmation by quantitative PCR and FISH mapping, complemented by Roche 454 sequencing of a microdissected B chromosome. They mapped those sequences to the scaffolds of the genome of the cichlid *Metriaclima zebra*, and detected blocks where the B+/B− coverage was higher than the average calculated for scaffolds not located in the B. Those blocks with high coverage in the B+ library where further annotated with the *M. zebra* genome. Among them, they found more than 5,000 sequences putatively identified as genes, besides repeats and transposable elements. Most of the genes found in the B chromosome were fragmented, but a few of them were complete and associated with functions such as microtubule organization, kinetochore structure, recombination and progression through the cell cycle, which may be involved in the transmission and maintenance of the parasitic chromosome^11^.

The general belief that B chromosomes are genetically inactive was enforced by experiments using tritiated uridine in the grasshoppers *Myrmeleotettix maculatus* and *Chorthippus paralelus*^12^, and in the rodent *Apodemus peninsulae*^13^. Nevertheless, gene expression in B chromosomes is probably behind the remarkable effects reported in some cases, such as the fungus *Nectria haematococca*, whose B chromosome carries a gene which confers resistance to pisatin, an antibiotic produced by the pea host plant^14^, or the paternal sex ratio (PSR) chromosome of the parasitic wasp *Nasonia vitripennis*, which causes the conversion of diploid zygotes (destined to be females) to haploid males^15^. More recently, it has been shown that B chromosomes influence sex determination in cichlid fishes^8^. Consistently, gene expression has recently been found in B chromosomes of several species. For instance, the presence of rRNA transcripts specifically coming from B chromosomes has been shown in the plant *Crepis capillaris*^16^, the parasitic wasp *Trichogramma kaykai*^17^ and the grasshopper *Eyprepocnemis plorans*^18^. Also, Carchilan *et al*. showed, in rye, the presence of B-specific transcribed DNA sequences belonging to high-copy number families with similarity to mobile elements^19^, while Zhou *et al*. characterized a B chromosome linked scaffold that contains an actively transcribed unit^20^, and Trifonov *et al*.reported the transcription of protein coding genes in *Capreolus pygargus*^9^. Recently, it has been reported that a repetitive element enriched in a B chromosome may code for a long non-coding RNA^21^ Thus, it has been suggested that B chromosome content might influence the expression of genes located in the standard A genome^19^,^21^,^22^,^23^. Indeed, genomic and transcriptomic analyses have revealed the presence of pseudogenic and functional copies of the Argonaute-like *AGO4B* gene on rye B chromosomes^4^.

The B chromosome system of *E. plorans* is very widespread and highly polymorphic, with Bs being present in almost all populations from the circum-Mediterranean region^24^. The presence of a same type of B in populations from Spain, Morocco, Tunisia and Sicilia suggests a recent invasion into these areas^25^. The high success of these B chromosomes, which are present in almost all populations hitherto analyzed, except those in the headwaters area of the Segura River basin in Spain^26^, has arisen from the transmission advantage (drive) they show during female meiosis^27^. This drive is suppressed by the A chromosomes^28^,^29^ as a consequence of the arms race between A and B chromosomes predicted by the near-neutral model of B chromosome evolution^30^. Neutralized B chromosomes (e.g. B2) can undergo chromosomal rearrangements (see examples in López-León *et al*.^31^), some of which might give rise to a new B-variant being able to drive (e.g. B24 in the Torrox population) thus replacing the neutralized variant^27^,^32^. Logically, this polymorphism regeneration prolongs very much the lifespan of the B chromosome polymorphism.

B chromosomes of *E. plorans* are able to transcribe their rDNA and organize a nucleolus^18^, although this occurs only in a minority of males from most populations^33^, and the rRNA contribution by the B to total rRNA is insignificant compared to that from A chromosomes^34^, suggesting that B chromosomes in this species are highly repressed. Here we analyze the possible presence of protein-coding genes in the B chromosome of this species by means of NGS analysis of 0B and 4B male genomes and their mapping against the coding sequence (CDS) regions of a *de novo* assembled transcriptome built with all the reads obtained from 0B and 1B female RNAs. This approach has revealed the presence of ten protein-coding genes, five of which are actively transcribed in males and females.

## Results

### At least ten protein-coding genes reside in the parasitic chromosome

Clustering and identification of potential CDSs in the *de novo* assembled *E. plorans* transcriptome yielded 13,190 sequences that were used as reference for mapping and comparative coverage analysis in 0B and 4B gDNA libraries (*E. plorans* transcriptome assembled *de novo* can be accessed in Figshare at https://dx.doi.org/10.6084/m9.figshare.3408580.v3).

**Figure 1:**
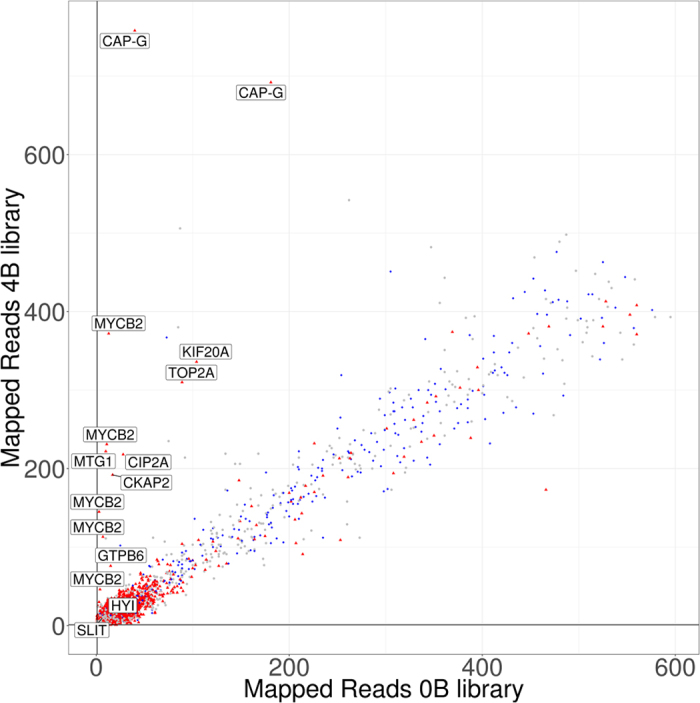
Identification of protein-coding genes located in B chromosomes of the grasshopper *E. plorans*, using the number of mapped reads that map to the CDSs found in the *de novo* assembled transcriptome, in the 0B (X axis) and 4B (Y axis) libraries of genomic DNA. Red triangles represent those sequences that code for known proteins, blue diamonds represent those sequences that code for transposable elements and grey circles represent non-annotated CDSs. The CDSs corresponding to the 10 genes found in the B chromosome are labelled.

The mapping of the Illumina reads from the 0B and 4B genomes on the coding sequences (CDS) of the *de novo* transcriptome revealed 2,592 CDSs with 40 or more mapped reads (considering both libraries). Graphical representation of the 0B and 4B libraries showed the presence of some CDSs being over-represented in the 4B library (Fig. 1). Remarkably, 29 CDSs showed a log2 4B/0B quotient >1.58, i.e. the expected value if each B chromosome carried one copy of the CDS (see materials and methods). Annotation revealed that 13 of these CDSs were orthologous to 8 different protein-coding genes in the *L. migratoria* genome^35^ (Table 1): *CIP2A* (CIP2A protein), *CKAP2* (Cytoskeleton-associated protein 2), *CAP-G* (Condensin I complex subunit G), *GTPB6* (GTP-binding protein 6), *KIF20A* (Kinesin-like protein KIF20A), *MTG1* (Mitochondrial GTPase 1), *MYCB2* (E3 ubiquitin-protein ligase MYCBP2), and *TOP2A* (DNA topoisomerase 2-alpha). In adition, for qPCR analysis, two other annotated CDSs were selected (*HYI* (Hydroxypiruvate isomerase) and *SLIT* (SLIT protein)) which, in a preliminary analysis, showed higher abundance in the 4B genome compared to 0B. The sequences of these ten transcripts can be found in GenBank under accession numbers KX034164 to KX034172 and KY211688 (Table 2).

**Figure 2:**
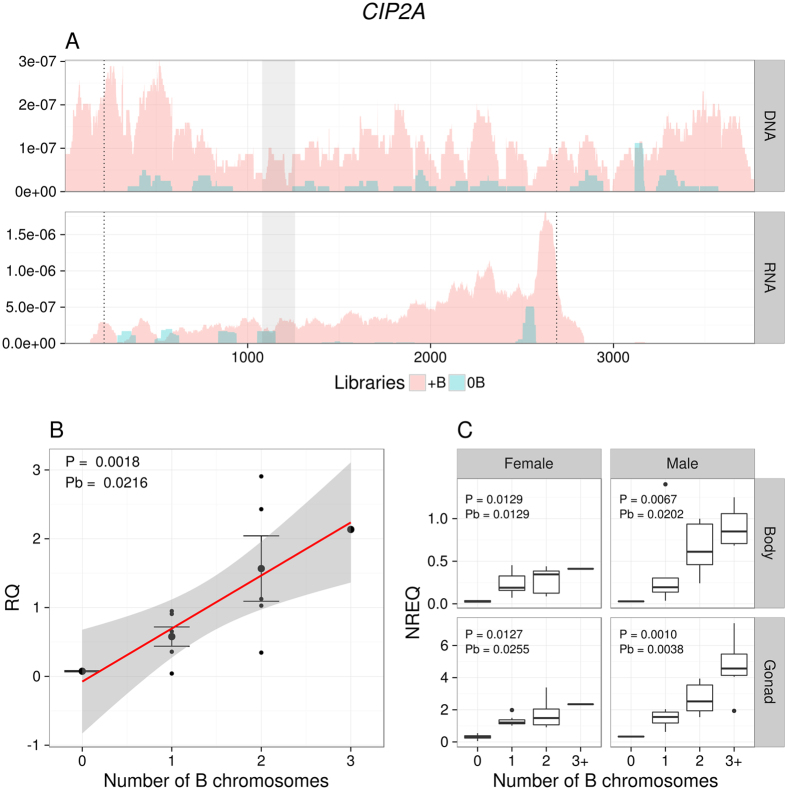
Coverage for the *CIP2A* transcript in the gDNA (0B and 4B) and RNA (0B and 1B) Illumina reads (**A**), and qPCR on gDNA (**B**) and cDNA (**C**). Note that coverage was higher in the 4B library across the complete sequence length, including the full CDS (delimited by the dotted vertical lines), the 5′ UTR (from the 5′ end to the first dotted line) and 3′ UTR (from the second dotted line to the 3′ end). Likewise, note the higher coverage for this transcript in the B-carrying RNA library. The shaded zone in A marks the region amplified by qPCR. qPCR on gDNA (**B**) revealed that genomic copy number for the *CIP2A* gene increases with B chromosome number, following a dose-dependent pattern, thus supporting its presence in the B chromosome. qPCR on cDNA (**C**) revealed that *CIP2A* is expressed in all tissues and sexes analyzed, also following a dose-depending pattern and suggesting the active transcription of B chromosome gene copies. RQ = Relative quantity. NREQ = Normalized relative expression quantity. P = P-value and Pb = Sequential Bonferroni P-value for Spearman rank correlation (**B**) and Kruskal-Wallis (**C**) analyses.

**Figure 3:**
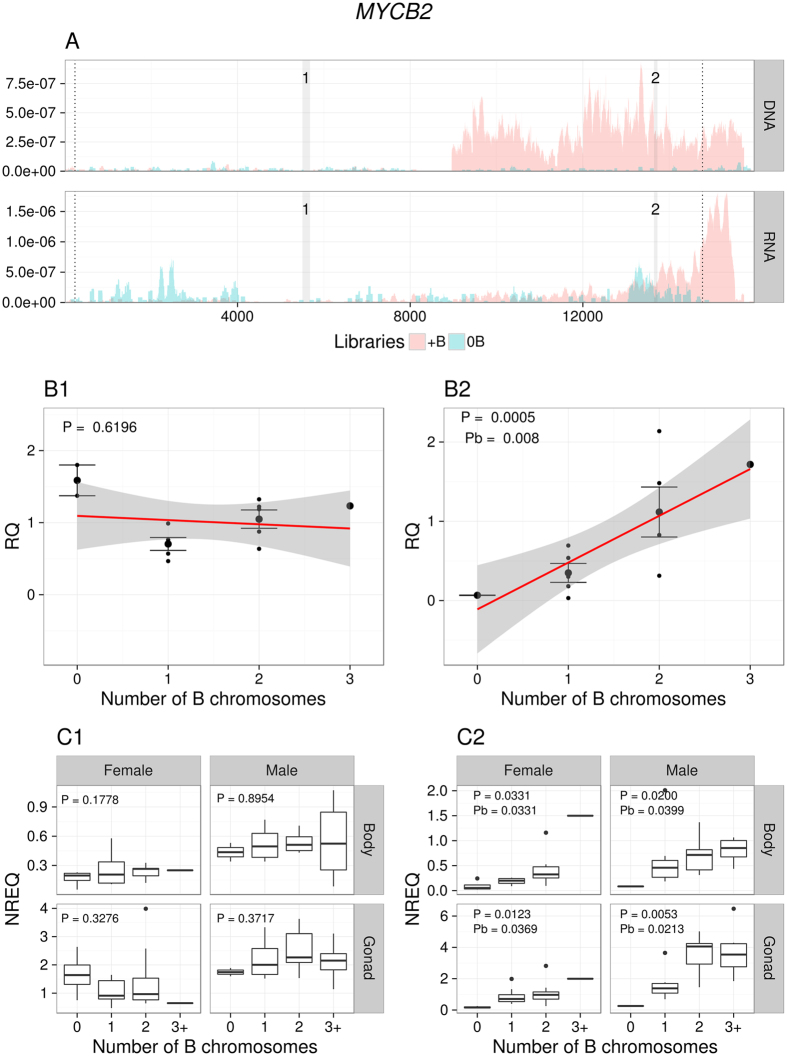
Coverage for the *MYCB2* transcript in the gDNA (0B and 4B) and RNA (0B and 1B) Illumina reads (**A**), and qPCR on gDNA (**B**) and cDNA (**C**). Note that less than half of *MYCB2* CDS (between the dotted vertical lines) showed high coverage in the 4B library, specifically from position 8961 to the 3′ end (**A**), suggesting that this B chromosome gene is truncated. Two regions were selected for qPCR amplification of this gene, one within the region being apparently absent in the B chromosome (shaded zone 1) and the other within the region being present in it (shaded zone 2). qPCR on gDNA with zone 1 primers showed that copy number for this gene region was independent on the number of B chromosomes (B1). However, qPCR on gDNA with zone 2 primers showed that abundance of this *MYCB2* gene region increased with B chromosome number in a dose-dependent pattern (B2). Likewise, qPCR on cDNA showed that MYCB2 expression was independent of B chromosome number when probed with zone 1 primers (C1) but it increased in a dosage-dependent pattern with zone 2 primers (C2), suggesting the active transcription of B chromosome truncated gene copies. RQ = Relative quantity. NREQ = Normalized relative expression quantity. P = P-value and Pb = Sequential Bonferroni P-value for Spearman rank correlation (**B**) and Kruskal-Wallis (**C**) analyses.

According to the coverage pattern observed in 0B and 4B genomes, full length CDSs were found in the 4B genome for four genes: *CIP2A, GTPB6, KIF20A* and *MTG1*, suggesting that these genes might be complete on the B chromosome. As shown in Fig. 2A and Supplementary Figs S1A, S2A and S3A, coverage for these genes in the gDNA 4B library was uniformly high along all CDS length. The six remaining genes (*MYCB2, CAP-G, CKAP2, HYI, SLIT* and *TOP2A*), however, appeared to be incomplete on the B chromosome. *MYCB2* was clearly truncated (Fig. 3A), showing only the last 5,764 nucleotides of the 3′ end out of the 14,434 nucleotides reported for the orthologous gene in *L. migratoria*. In *CAP-G*, the last five exons (exons 20–24) of the CDS reported for the *L. migratoria* genome show low coverage in the *E. plorans* 4B genome (Supplementary Fig. S4A). In *CKAP2*, the 5′ UTR and 396 nucleotides of the 5′ end of the CDS seem to be missing (Supplementary Fig. S5A). For *HYI*, we only observed 85 nucleotides in the 3′ end of the CDS plus the 3′ UTR, but this gene actually showed very low coverage in both gDNA libraries, and its integrity was difficult to assess (Supplementary Fig. S6A). In the case of the *SLIT* gene, only the last two exons of the 5′ end were in the B chromosome, out of the 30 exons reported in *L. migratoria* (Supplementary Fig. S7A). Finally, *TOP2A* coverage in the 4B library was low from nucleotide 3,465 to the 3′ end of the transcript, suggesting that this part is not present in the B chromosome copies (Supplementary Fig. S8A).

On the basis of mean abundances per nucleotide position in 0B and 4B libraries (*A*_0*B*_ and *A*_4*B*_, respectively) inferred from mapping Illumina gDNA reads onto transcriptome CDS regions, and assuming that these ten genes are single-copy in the A chromosome set, we estimated the number of copies in the B-carrying genome (*N*_4*B*_) and also per B chromosome (*N*_*B*_). This suggested the presence of two or more copies for all ten genes in the B chromosome, with *N*_*B*_ ranging from 2 to 17 (Table 2).

qPCR experiments on gDNA from males carrying 0–3 B chromosomes showed that genomic abundance for these ten genes increased linearly with the number of B chromosomes (Figs 2B and 3B, Supplementary Table S1 and Supplementary Figs S1B–S7B), thus giving high support to the NGS results. Remarkably, in the six truncated genes (*MYCB2, CAP-G, CKAP2, HYI, SLIT* and *TOP2A*) this linear relationship was observed only in the region showing high coverage in the gDNA 4B library, implying their location in the B chromosome, while no relationship with B number was observed for the low coverage region thus supporting its absence on the B chromosome and the existence of fragmented B chromosome gene copies (see Fig. 3B and Supplementary Figs S4B–S8B).

### The parasitic chromosome is transcriptionally active

The analysis of differential gene expression between B-carrying and B-lacking individuals, by means of qPCR, revealed that five out of the ten genes located on the B chromosome (*CIP2A, CKAP2, CAP-G, KIF20A*, and *MYCB2*) showed significant up-regulation in B-carrying males (all genes) and females (all except KIF20A) (Supplementary Table S2), suggesting that some of the B-located gene copies are transcribed. Remarkably, three of these genes (*MYCB2, CAP-G* and *CKAP2*) are truncated on the B chromosome and showed differential expression only for the gene regions contained in the B chromosome but not for missing regions (see Figs 3C, S4C and S5C, and Supplementary Table S2). This strongly supports that the up-regulation of these genes is due to the activity of the B chromosome copies and not simply to up-regulation of the A chromosome gene copies.

For *CIP2A* (Fig. 2C) and *KIF20A* (only in males, Supplementary Fig. S2C) genes, expression level increased with B chromosome number, and a similar dosage effect was observed for *MYCB2* (Fig. 3C), *CAP-G* (Supplementary Fig. S4C) and *CKAP2* (Supplementary Fig. S5C) in the case of gene regions being present in the B chromosome, whereas, in *TOP2A*, this association was only marginally significant (Supplementary Fig. S8C). No significant differential expression was found for the gene regions being missing in the B chromosome for *MYCB2* (Fig. 3C), *CAP-G* (Supplementary Fig. S4C), *CKAP2* (Supplementary Fig. S5C) and *TOP2A* (Supplementary Fig. S8C), or for the *GTPB6* (Supplementary Fig. S1C), *MTG1* (Supplementary Fig. S3C), *HYI* (Supplementary Fig. S6C) and *SLIT* (Supplementary Fig. S7C) genes. Taken together, these results reinforce the conclusion that about half of the B chromosome genes identified here are actively transcribed.

Gene Ontology (GO)^36^ analysis for these ten genes revealed potential implication in biological processes likely profitable for a parasitic B chromosome, such as the regulation of mitotic cell cycle (*KIF20A, CAP-G*, and *CKAP2*), DNA replication and regulation of transcription (*CKAP2* and *MYCB2*), apoptotic processes and regulation of cell death (*CKAP2*), chromosome condensation and organization (*CAP-G* and *TOP2A*), chromosome segregation (*CAP-G* and *TOP2A*), cell-cell signaling and cellular response to stimulus (*SLIT*), and reproductive structure development (*SLIT*) (see Supplementary Dataset 1). The EuKaryotic Orthologous Groups (KOG)^37^ classification of these ten genes also gave interesting indications of their potential functions (Table 3), some of which being highly valuable for the advantageous transmission of this parasitic chromosome. For instance, *CIP2A* and *KIF20A* have functions related with cytoskeleton and thus microtubule dynamics, *CAP-G* and *TOP2A* are related with chromosome condensation and chromatin structure and dynamics, and thus with cell cycle control. The two former genes appear to be complete and active in the B chromosome, but the two latter are truncated thus probably rendering non-translated or inactive transcripts.

## Discussion

As intranuclear parasites, B chromosomes mimic A chromosomes in many respects, for example the structure and organization of the DNA sequences contained in them, although they are usually heterochromatic and, as such, assumed to be genetically inert elements (for review, see Camacho^2^).

Recently, transcription of a protein-coding gene on B chromosomes of the Siberian roe deer (*Capreolus pygargus*) has been shown^9^, while Banaei-Moghaddam *et al*. used NGS to demonstrate that about 15% of the pseudogene-like fragments on B chromosomes are transcribed following a pattern related to genotype and tissue type, with some of them apparently playing a role in trans-regulation of genes located in the A chromosomes^22^. Likewise, Valente *et al*. analyzed transcriptome sequences from the cichlid fish, *Pundamilia nyererei*, and some of them showed high sequence similarity with the B-encoded variants for the *Separin, TUBB1* and *KIF11* genes found in *Astatotilapia latifasciata*, thus suggesting that *P. nyererei* might have B chromosomes expressing these genes^11^. These findings clearly contradict the “B genetic inertness” hypothesis, although whether B chromosomes express truly functional genes (i.e. generate proteins) or regulatory factors (i.e. small RNAs) remains to be tested. Recently, Ma *et al*. have shown that rye B chromosomes carry active Argonaute-like genes showing *in vitro* slicer activity, thus opening the interesting prospect that B chromosomes may carry functional genes influencing other genes’ expression or cellular processes^4^. However, the only B chromosome genes which have been shown to yield functional transcripts *in vivo* are those for rRNA in the grasshopper *E. plorans*, as they give rise to the expected phenotype, i.e. a nucleolus^18^.

Here we show that B chromosomes in *E. plorans* contain at least ten protein-coding genes, with full CDS regions in four of them. The qPCR validation of two genes failing to meet the *a priori* selection criteria of the NGS analysis (i.e. *HYI* and *SLIT*) demonstrated a positive correlation with number of B chromosomes, indicating that the NGS selection criteria are actually conservative and that these B chromosomes most likely carry other undetected genes. Remarkably, five of these ten genes are actively transcribed in *E. plorans*. Many of the B-derived transcripts might be functionless because the gene copies on the B chromosome are incomplete, so that their translation would yield anomalous polypeptides which could potentially pose metabolic stress on cells. Alternatively, these gene fragments could interfere A chromosome gene expression by competitively binding transcription factors^38^,^39^,^40^. However, we cannot rule out that the transcripts from some B-located genes, being apparently complete, can be functional, as previously observed for 45S rRNA transcripts^18^. Of course, the possibility that a B chromosome can contribute gene products which are useful for its own survival (e.g. through cell division) is a new and interesting prospect in B chromosome research.

Three genes which are truncated on the B chromosome (*CKAP2, CAP-G* and *MYCB2*) show up-regulation only for the region being present in the B chromosome, indicating that gene copies located on the B chromosome are transcribed, although our data cannot differentiate this from a more complex transcriptional regulatory pathway involving A and B copies. *CKAP2* codes for a cytoskeleton-associated protein which localizes to spindle poles and microtubules from prophase to anaphase^41^, and seems to play an important role in chromosome segregation and stability^42^,^43^. *CAP-G* codes for a subunit of the Condensin I chromosome condensation complex, subunit G^44^, whereas *MYCB2* encodes a component protein of the anaphase promoting complex (APC) governing the exit from mitosis^45^. If these transcripts were translated, they would presumably yield non functional truncated polypeptides, especially *MYCB2*, where more than half of the CDS is missing in the B chromosome. It is however tempting to speculate whether the strong functional relationship between *CAP-G* and *TOP2A* in sister chromatid resolution^46^ and chromosome condensation^47^ might have played a role in the invasion of the *E. plorans* genome by this B chromosome, in the event that these genes were ever complete and functional in it.

It is also conceivable that, as suggested by Banaei-Moghaddam *et al*., these gene fragments present in the B could act as trans modulators, affecting the activity of its counterparts located in the A genome^22^. A possible example of this could be the down-regulation shown by the *GTPB6* gene in the 1B RNA library (see Supplementary Fig. S1A), meaning that it would be repressed in presence of the B chromosome, and a similar trend was observed in qPCR experiments on ovary (Supplementary Fig. S1C), although it was not significant (Supplementary Table S2).

Remarkably, the two remaining up-regulated protein-coding genes (*CIP2A* and *KIF20A*), which were complete in the B chromosome, coded for potentially interesting functions for a parasitic chromosome. *CIP2A* codes for an oncoprotein that inhibits protein phosphatase 2A (*PP2A*), promoting anchorage-independent cell growth and tumor formation, and its overexpression causes premature chromosome segregation and aneuploidy^48^. Bearing in mind that B chromosomes most likely originate as a kind of aneuploidy^2^,^49^, it is conceivable that the excess of *CIP2A* gene products, provided by transcription from the B chromosome copies, might be advantageous for B chromosome maintenance. Likewise, *KIF20A* codes for a mitotic kinesin required for chromosomal passenger complex (CPC) transport during cytokinesis^50^ and for appropriate assembly of microtubules at anaphase and metaphase-anaphase transition^51^. In Xenopus, Takemoto *et al*. showed that PP2A plays a role in the recruitment and targeting of Condensin II and kinesin protein KIF4a to chromosomes during mitosis, and *PP2A* inhibition causes Condensin II and KIF4a dissociation from assembled chromosomes^52^. In *E. plorans*, B chromosomes carrying active *CIP2A* and *KIF20A* genes could potentially influence the course of cell division for their own benefit, thus revealing their true parasitic nature. In rye, the presence of repetitive DNA sequences in the short arm of the B chromosome promotes mitotic nondisjunction which is the basis for its drive mechanism^53^. In *E. plorans*, B chromosome drive takes place during female meiosis^27^,^28^, and the possibility of manipulating it through gene expression might be the basis for the high success of B chromosomes in this species, as they are present in almost all natural populations hitherto analyzed^24^.

Taken together, our results show, for the first time, that the secret for B chromosome success may lie in its gene content, as suggested by the active transcription of the complete CDS of *CIP2A* and *KIF20A* and the fragments of *CKAP2, CAP-G* and *MYCB2*, all being genes with functions related with cell division. Interestingly, the presence of this kind of active genes in *E. plorans* B chromosomes opens new avenues to investigate why neutralized B variants are replaced for newly driving ones, a fact reported several times in this species^27^,^30^,^32^. The possibility that a chromosomal rearrangement in a neutralized B chromosome can elicit position effects changing the expression state of B chromosome genes with functions related with cell division, can now be investigated by comparing gene expression levels for B-genes between different B-variants.

## Methods

### Materials

*E. plorans* individuals were collected in Torrox (Málaga) (Table 4), a population where the prevalent B chromosome variant is B24^25^. Males were anaesthetized before dissecting out testes, one of which was fixed in 3:1 ethanol-acetic acid for cytological analysis, while the other testis and body remains were frozen in liquid nitrogen for nucleic acid extraction. The number of B chromosomes in males was determined by C-banding of testicular follicles. In the case of the two females used for transcriptome analysis, the number of B chromosomes was determined in interphase hemolymph nuclei^54^. The full bodies of the two females selected for transcriptome analysis (one 0B and one with 1B) were frozen in liquid nitrogen. The remaining females were anaesthetized before dissecting out the ovaries. A few ovarioles were incubated in 2% colchicine in isotonic insect saline solution for 2h, and then fixed in 3:1 ethanol-acetic acid for cytological analysis. The remaining ovarioles were frozen in liquid nitrogen for gene expression analysis. Body remains were frozen in liquid nitrogen for later nucleic acid extraction. In these females, the number of B chromosomes was determined by C-banding on the colchicine-treated ovarioles. Additionally, B chromosome presence/absence was corroborated in both sexes by PCR amplification of the B-specific SCAR marker, described by Muñoz-Pajares *et al*.^55^, on genomic DNA.

### Nucleic acid isolation

Genomic DNA (gDNA) was extracted from hind legs using the GenElute Mammalian Genomic DNA Miniprep kit (Sigma). Quality was checked by TBE-agarose gel electrophoresis and also by measuring 260/280 and 260/230 ratios with an Infinite M200 Pro NanoQuant (Tecan). Total RNA extractions from frozen bodies were performed using a Real Total RNA Spin Plus kit (Durviz), whereas RNA extractions from gonads were done using the RNeasy Lipid Tissue Mini Kit (Qiagen), following manufacturer’s recommendations. In both protocols, we carried out a DNAse treatment on the column membrane (20 units during 30 minutes incubation, DNAse Amplification Grade I, Sigma), to avoid gDNA contamination, which was validated by PCR amplification of rDNA or histone genes in the extracted RNA and subsequent visualization on an agarose gel. In body samples, a second DNAse treatment was performed after the extractions, using the REALSTAR kit (Durviz). Quality and quantity of RNA was assessed with a Tecan’s Infinite 200 NanoQuant spectrophotometer and in a denaturing MOPS-agarose gel to ensure the absence of RNA degradation.

### Illumina sequencing

Total RNA was extracted from each of two females, one lacking B chromosomes (0B) and the other carrying 1B, whereas gDNA was collected from two males (0B and 4B; Table 4). Each of the four libraries (gDNA 0B, gDNA 4B, RNA 0B, RNA 1B) was sequenced on an Illumina Hiseq2000 platform, each yielding about 5 Gb of paired-end reads (2 × 101 nucleotides). Illumina sequences are available in NCBI SRA database under accession numbers SRR2970625 (gDNA 0B), SRR2970627 (gDNA 4B), SRR2969416 (RNA 0B) and SRR2969417 (RNA 1B).

### Analysis of abundance and integrity of protein-coding genes located putatively in the B chromosome

We used an *E. plorans de novo* transcriptome^35^ asembled using Trinity^56^, as reference for read mapping. We reduced redundancies with CD-HIT-EST^57^ with local alignment and greedy algorithm, and grouped those sequences showing 80% or higher similarity in at least 80% of length (options -M 0 -aS 0.8 -c 0.8 -G 0 -g 1). Potential CDSs were predicted with Transdecoder^56^, considering open reading frames (ORFs) longer than 300 nucleotides. Functional annotation of CDSs was performed with Trinotate^56^ against the Uniprot database^58^, and the identification of transposable elements (TEs) within the CDS was performed with RepeatMasker^59^ in a database including TEs described in *Locusta migratoria* (data obtained from RepBase^60^).

To search for protein-coding genes residing in the B chromosome, we performed the following analysis. Against the *de novo* transcriptome, we mapped: 1) the reads obtained from the 0B and 4B genomes (gDNA 0B and gDNA 4B libraries), and 2) those obtained from RNA sequencing in 0B and 1B females (RNA 0B and RNA 1B libraries). We mapped the reads using SSAHA2^61^ with a minimum alignment score of 40 and 80% minimum identity. We used a custom script (https://github.com/fjruizruano/ngs-protocols/blob/master/count_reads_bam.py) to count the number of mapped reads as a measure of abundance (see a graphical summary of analysis workflow in Supplementary Fig. S9).

We selected coding sequences (CDS) putatively being located in the B chromosome on the basis of the two following criteria: 1) the sum of mapped reads (adding those from 0B and 4B gDNA libraries) should be 40 or higher, and 2) log_2_ of the quotient between the number of mapped reads in the 4B and 0B gDNA libraries (4B/0B) was equal to or higher than 1.58. This figure was inferred by assuming that a single-copy gene would have two copies in a diploid 0B genome, whereas, if each B chromosome would carry one copy then the 4B genome would carry six copies, i.e. three times more copies than the 0B one, so that log_2_ (3) = 1.58.

We analyzed the integrity and structure of the B-located genes using a transcriptome assembly from 12 RNA-Seq libraries of *Locusta migratoria* (Ruiz-Ruano *et al*., personal communication) and the *L. migratoria* genome assembled by Wang *et al*. (accession number AVCP000000000)^35^. We searched for homologous sequences in the *L. migratoria* transcriptome with BLASTN^62^. Using the sequence with the lowest E-value as reference for a second BLASTN^62^, we searched for homologous sequences in the *L. migratoria* genome. We aligned our transcripts from *E. plorans* to the selected genomic sequences with the exonerate software^63^ to search for exon junction sites. If an *E. plorans* transcriptome sequence was incomplete with respect to the *L. migratoria* one, we used the homologous contig of *L. migratoria* transcriptome as reference for further assembling of the *E. plorans* contigs, to full-length *E. plorans* transcripts wherever possible. Using them as reference, we performed a new SSAHA2^62^ mapping to test the completeness of the CDSs of these genes in the B chromosome. We thus analyzed abundance per nucleotide position for each CDS in the 0B and 4B genomes using a custom script (https://github.com/fjruizruano/ngs-protocols/blob/master/bam_coverage_join.py), and calculated it as the proportion of reads mapped at each position, in respect to total number of reads in the corresponding library. This allowed detecting whether a CDS was completely covered by the B-carrying gDNA reads, thus implying its full length presence in the B chromosome. Alternatively, a sharp decrease in nucleotide coverage along CDS length indicated truncation for the corresponding gene in the B chromosome. For full CDSs, we calculated the mean abundance for its whole length in 0B and 4B gDNA libraries. In the case of truncated CDSs, we calculated mean abundance for the highly covered region.

Mean abundance for each CDS (named *A*_0*B*_ and *A*_4*B*_ in 0B and 4B males, respectively) was used to estimate the number of copies for the corresponding gene in the B-carrying genome, assuming the presence of one copy per A genome set. For this purpose, we first calculated genome size of 0B and 4B males. According to Ruiz-Ruano *et al*., the *E. plorans* genome is 1.78 times larger than that of *Locusta migratoria*^64^. On this basis these authors got estimates of DNA content of A and B chromosomes in *E. plorans*, using previous estimates of C.value in *L. migratoria* (5.89 pg = 5.76 Gb). However, Wang *et al*. later showed that the sequenced genome of this latter species is actually larger (6.3 Gb)^35^, for which reason we have recalculated, in *E. plorans*, the DNA amount in the haploid A chromosome set (C-value), and those in the X and B24 chromosomes, being 11.214 Gb, 1.352 Gb and 0.684 Gb, respectively. Bearing in mind that males of this species are X0, we calculated genome size in the 0B and 4B males (*G*_0*B*_ = 20.58 Gb and *G*_4*B*_ = 23.26 Gb, respectively).

The number of gene copies in the B-carrying genome (*N*_4*B*_) was then calculated by the following equation:


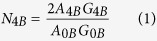


where 2 means assuming the presence of one gene copy per A chromosome set. The number of gene copies per B chromosome (*N*_*B*_) was then calculated as:


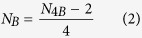


where 4 is the number of B chromosomes in the B-carrying male.

Gene function classifications were performed according to two standardized methods: Gene Ontology (GO)^36^ and Eukaryotic Orthologous Groups (KOG)^37^. GO assignments to predicted proteins were performed with Blast2GO^65^, and KOG classification was performed with the WebMGA software^66^, searching the KOG database of NCBI.

### Relative quantification of genomic abundance and transcription analysis of the ten protein-coding genes residing in the B chromosome

Quantification of relative copy number for the B chromosome genes was performed in 16 males from Torrox carrying 0–3 B chromosomes (Table 4). Transcription of B-located genes was analyzed in 23 males and 21 females from Torrox (Table 4), separately in the gonads and the rest of the body. cDNA synthesis was performed by retro-transcription of 100 ng per sample of total RNA using a combination of random and oligo dT hexamers (PrimeScript RT reagent Kit, Perfect Real Time, Takara). The cDNA obtained was diluted in RNase-DNase free water for a 1:10 working solution.

Quantitative PCR was performed on a Chromo 4 Real Time PCR thermocycler (Biorad). Primers were designed with Primer3^67^ (see sequences in Table 5). Each reaction mixture contained 5 μl of gDNA at 5 ng/μl (25 ng gDNA per reaction) or 5 μl of cDNA working solution obtained as described above, 5 μl of SensiMix SYBR Kit (Bioline) and 2.5 μl of each 2.5 μM primer. Reactions were carried out in duplicate and the coefficient of variation was lower than 8% in all cases. We estimated the amplification efficiency (E) of each primer pair in gDNA or cDNA experiments by means of a standard curve performed on a 10-fold dilution series of *E. plorans* gDNA or cDNA mixture from several individuals with different numbers of B chromosomes, which was also used as an external calibrator. Then the relative abundance of each gene in 0B, 1B, 2B and 3B genomes was calculated according to RQ = E^CtC−CtS^. where RQ = Relative quantity, E = Amplification efficiency (fold increase per cycle), CtC = Ct value of the calibrator sample and CtS = Ct value of each sample.

RQs of the expression analysis were calculated according to the same formula, but RQ values were normalized by the geometrical average of several housekeeping genes, i.e. Ribosomal Protein 49 (*RP49*), Actin 5C (*ACT*), Armadillo (*ARM*), and α-tubulin 1A (*TUB*), selected using GeNorm^68^. We used *RP49*+*ACT*+*ARM* for female bodies, *ACT*+*ARM* for ovaries, *ACT*+*TUB* for male bodies and *ACT*+*RP49* for testis. Amplification, sequencing, efficiency and stability analysis of these housekeeping genes in *E. plorans* was previously performed in Navarro-Domínguez *et al*.^69^

### Statistical analysis

For qPCR validation of gene presence in the B chromosome, we should expect that those genes actually residing in the B chromosome would show RQ values on gDNA linearly increasing with the number of B chromosomes. This relationship was tested by the Spearman’s rank correlation test. Differential gene expression was tested by means of Kruskal-Wallis tests comparing individuals with different number of B chromosomes. In all cases, the sequential Bonferroni test was applied to minimize type I errors.

## Additional Information

**How to cite this article:** Navarro-Domínguez, B. *et al*. Protein-coding genes in B chromosomes of the grasshopper *Eyprepocnemis plorans. Sci. Rep.* **7**, 45200; doi: 10.1038/srep45200 (2017).

**Publisher's note:** Springer Nature remains neutral with regard to jurisdictional claims in published maps and institutional affiliations.

## Supplementary Material

Supplementary Information

Supplementary Dataset 1

## Figures and Tables

**Table 1:** Results of mapping gDNA reads from 0B and 4B males on transcriptome CDSs.

Transcriptome CDS	Length	gDNA reads mapped		Log2(4B/0B)	Annotation
		0B	4B		
comp59256_c3_seq2|m.36041	468	3	211	6.14	TE
comp61215_c1_seq1|m.53202	519	3	145	5.59	*MYCB2*
comp61215_c0_seq1|m.53195	906	13	372	4.84	*MYCB2*
comp60327_c0_seq1|m.44236	966	10	222	4.47	*MTG1*
comp61215_c0_seq1|m.53194	1338	11	231	4.39	*MYCB2*
comp62628_c0_seq2|m.69037	3012	40	758	4.24	*CAP-G*
comp61215_c5_seq1|m.53215	369	7	113	4.01	*MYCB2*
comp59256_c1_seq2|m.36039	441	19	250	3.72	NA
comp61215_c2_seq1|m.53203	684	4	46	3.52	*MYCB2*
comp62255_c0_seq1|m.65104	1767	17	192	3.50	*CKAP2*
comp59256_c1_seq1|m.36038	324	10	111	3.47	NA
comp59256_c0_seq1|m.36037	351	6	58	3.27	NA
comp59183_c0_seq1|m.35620	2367	28	218	2.96	*CIP2A*
comp43869_c0_seq1|m.5004	1098	215	1492	2.79	TE
comp40101_c0_seq1|m.3160	411	87	506	2.54	NA
comp61379_c0_seq1|m.55345	1653	15	76	2.34	*GTBP6*
comp57756_c0_seq1|m.28314	357	73	367	2.33	TE
comp57756_c0_seq1|m.28313	972	286	1415	2.31	TE
comp40101_c0_seq1|m.3162	315	85	380	2.16	NA
comp62313_c1_seq11|m.65571	351	44	180	2.03	NA
comp61143_c1_seq6|m.51252	837	25	102	2.03	TE
comp62628_c0_seq8|m.69051	1728	181	692	1.93	*CAP-G*
comp62313_c1_seq1|m.65560	498	324	1174	1.86	NA
comp62453_c1_seq3|m.66898	4269	89	310	1.80	*TOP2A*
comp52884_c0_seq1|m.14126	651	235	780	1.73	NA
comp57756_c1_seq1|m.28315	1374	605	1969	1.70	TE
comp62575_c1_seq4|m.68249	2322	104	336	1.69	*KIF20A*
comp46268_c0_seq2|m.7460	339	320	1017	1.67	NA
comp62313_c1_seq11|m.65572	312	75	235	1.65	NA

Only the 29 contigs with 40 or more reads mapped and log2(4B/0B) ≥ 1.58 are shown, 13 of which corresponded to protein-coding genes, 6 to transposable elements (TE) and 10 failed to be annotated (NA).

**Table 2:** Estimation of the number of gene copies in the B24 chromosome of *E. plorans*.

Gene	Acc.No.	*A*_0*B*_	*A*_4*B*_	*N*_4*B*_	*N*_*B*_	B-activity
*CIP2A*	KX034164	0.0127	0.1161	21	5	Yes
*GTPB6*	KX034167	0.0099	0.054	12	4	No
*KIF20A*	KX034169	0.0484	0.2057	10	2	Yes
*MTG1*	KX034170	0.0123	0.3313	61	12	No
*CKAP2*	KX034165	0.0139	0.1582	26	8	Yes
*CAP-G*	KX034166	0.0137	0.2856	47	12	Yes
*HYI*	KX034168	0.0073	0.0814	25	4	No
*MYCB2*	KX034171	0.0103	0.3235	71	23	Yes
*SLIT*	KX034172	0.0117	0.0608	12	3	No
*TOP2A*	KY211688	0.0218	0.1088	11	2	No

The first four genes were found complete in the B-carrying genome, and the latter six were truncated. Acc.No. = Accession number (GenBank). Abundances in the 0B and 4B genomes (*A*_0*B*_ and *A*_4*B*_) are multiplied by 10^6^. *N*_4*B*_ = Number of copies in the 4B genome. *N*_*B*_ = Number of copies per B chromosome.

**Table 3:** KOG classification of genes located on the B chromosome.

Gene	Hit	E-Value	Description	Mult/Single	Class	Class Description
*CAP-G*	KOG2025	7.00E-31	Chromosome condensation complex Condensin I, subunit G	Multiple	B/D	Chromatin structure and dynamics/Cell cycle control and mitosis
*CIP2A*	KOG0161	4.00E-10	Myosin class II heavy chain	Single	Z	Cytoskeleton
*GTPB6*	KOG0410	2.00E-85	Predicted GTP binding protein	Single	R	General function prediction only
*HYI*	KOG4518	2.00E-75	Hydroxypyruvate isomerase	Single	G	Carbohydrate transport and metabolism
*KIF20A*	KOG0247	3.00E-84	Kinesin-like protein	Single	Z	Cytoskeleton
*MTG1*	KOG2485	4.00E-86	Conserved ATP/GTP binding protein	Single	R	General function prediction only
*MYCB2*	KOG1428	7.00E-84	Neuronal presynaptic protein Highwire/PAM/RPM-1	Single	T	Signal transduction mechanisms
*SLIT*	KOG4237	0.00	Extracellular matrix protein slit, contains leucine-rich and EGF-like repeats	Multiple	W	Extracellular structures/Signal transduction mechanism
*TOP2A*	KOG0355	0.00	DNA topoisomerase type II	Single	B	Chromatin structure and dynamics

*CKAP2* did not achieve any significant hits in KOG database.

**Table 4:** Biological samples used for each experiment in the current work.

Experiment	Technique	Sex	Body part	Bs	N
B chromosome gene content	Illumina WGS	Male	Hind leg	0	1
				4	1
	qPCR (DNA)	Male	Body	0	2
				1	6
				2	5
				3	1
B chromosome expression	Illumina RNA-seq	Female	Full body	0	1
				1	1
	qPCR (cDNA)	Male	Body/Testes	0	2
				1	7
				2	8
				3	6
		Female	Body/Ovary	0	4
				1	8
				2	8
				3	1

Bs = number of B chromosomes; N = number of individuals.

**Table 5:** Sequence of all primers used for qPCR experiments in this work.

Gene	Forward primer (5′-3′)	Reverse primer (5′-3′)
*CIP2A*	TGGCGCTGGTACTGAGTATG	GATCCACCTGAAGAGCTTGG
*CKAP2_1*	CAAAATGGCGTGCTGAAAG	CGTCTTTTGATTTAATAGTGGAATTTG
*CKAP2_2*	TCTTCGATGTTTTGGCCTTC	TGGTCATCATTTGCCAGAGA
*CAP-G_1*	GAGGTATGGAACACGCACAA	AGTGGCACGTTTCGTCTTCT
*CAP-G_2*	CAACAGCGCCTGTCACTAAA	GCTGAGGTGTCTGCTCACAA
*GTPB6*	CACTGGAGGACGCGATGT	CCGGAGTGAGATCAAAAGACC
*HYI_1*	TGTCCGGACGAGTTGACA	CGGAACAGAATGTGGATTGA
*HYI_2*	CCACATCCAGATTGCACAAG	ACTCCAAACCAATCCAACCA
*KIF20A*	CAGGGCACAAATGAAAATCC	TTGCTGCTTCTCTTCATCCA
*MTG1*	AGCTCCAGTAGGTGCAAAGG	GGCCTGCTTCAACATCTCT
*MYCB2_1*	ACCCGTCACATACACAACGA	CCATCACCATTGCTTGTACG
*MYCB2_2*	GCAAGGAAGAAGAGGAAGCA	CCAGTGCCATAACCCAGAAC
*SLIT1*	AACATGCTGCATTGCGACT	CACTTGAAGTCGTGGTCGTG
*SLIT2*	AACCTGTCGGAAAGAGCAAA	TTCGCACAATGTCAACATCC
*TOP2A_1*	GGAACACTCGGCGTACCA	GCAGAGTCCTTCCCACCA
*TOP2A_2*	GCCTGTCAAGGGCAAGAA	TCCAACTTCGGAGCAACC
