# Supplementary material for: Protein-coding genes in B chromosomes of the grasshopper Eyprepocnemis plorans
Source: Sci Rep. 2017 Apr 3;7:45200. doi: 10.1038/srep45200 (PMC5377258; doi:10.1038/srep45200)
Supplement: Supplementary Information [file srep45200-s1.pdf]

Supplementary Information

Protein-coding genes in B chromosomes of the grasshopper

Eyprepocnemis plorans

Beatriz Navarro-Domínguez[1], Francisco J. Ruiz-Ruano[1], Josefa Cabrero[1], José María Corral[2,3], María Dolores López-León[1], Timothy F. Sharbel[2,4], Juan Pedro M. Camacho[1*]

[1] Departamento de Genética, Facultad de Ciencias, Universidad de Granada (Spain)

[2] Leibniz Institute for Plant Genetics and Crop Plant Research (IPK), Gatersleben (Germany)

[3] Department of Bioanalytics, Coburg University of Applied Sciences and Arts (Germany)

[4] Global Institute for Food Security (GIFS), University of Saskatchewan (Canada)

* Corresponding author: jpmcamac@ugr.es

Contents

Supplementary Tables **3**

Table S1 | Spearman rank correlation (rS) between genomic abundance of nine genes and the number of B chromosomes, found in qPCR experiments.. 3

Table S2 | Kruskal-Wallis analysis of qPCR gene expression experiments in males and females with 0-3 B chromosomes.. 4

Supplementary Figures **6**

Figure S1 | Coverage for the *GTPB6* transcript in the gDNA (0B and 4B) and RNA (0B and 1B) Illumina reads (A), and qPCR on gDNA (B) and cDNA (C).................... 6

Figure S2 | Coverage for the *KIF20A* transcript in the gDNA (0B and 4B) and RNA (0B and 1B) Illumina reads (A), and qPCR on gDNA (B) and cDNA (C).................... 7

Figure S3 | Coverage for the *MTG1* transcript in the gDNA (0B and 4B) and RNA (0B and 1B) Illumina reads (A), and qPCR on gDNA (B) and cDNA (C).................... 8

Figure S4 | Coverage for the *CAP-G* transcript in the gDNA (0B and 4B) and RNA (0B and 1B) Illumina reads (A), and qPCR on gDNA (B) and cDNA (C).................... 9

Figure S5 | Coverage for the *CKAP2* transcript in the gDNA (0B and 4B) and RNA (0B and 1B) Illumina reads (A), and qPCR on gDNA (B) and cDNA (C).................... 11

Figure S6 | Coverage for the *HYI* transcript in the gDNA (0B and 4B) and RNA (0B and 1B) Illumina reads (A), and qPCR on gDNA (B) and cDNA (C).................... 13

Figure S7 | Coverage for the *SLIT* transcript in the gDNA (0B and 4B) and RNA (0B and 1B) Illumina reads (A), and qPCR on gDNA (B) and cDNA (C).................... 15

Figure S8 | Coverage for the *TOP2A* transcript in the gDNA (0B and 4B) and RNA (0B and 1B) Illumina reads (A), and qPCR on gDNA (B) and cDNA (C).................... 17

Figure S9 | Workflow of analysis... 19

Supplementary Tables

Gene	N	r S	P	Pb
CIP2A	14	0.7557	0.0018	**0.0216**
CKAP2_1	14	0.3755	0.1858	
CKAP2_2	14	0.7815	0.0010	**0.0150**
CAP-G_1	14	0.744	0.0023	**0.0230**
CAP-G_2	14	0.2065	0.4787	
GTPB6	14	0.7698	0.0013	**0.0182**
HYI_1	14	0.1432	0.6254	
HYI_2	14	0.7581	0.0017	**0.0221**
KIF20A	14	0.6008	0.0231	0.1617
MTG1	14	0.7416	0.0024	**0.0216**
MYCB2_1	14	-0.1455	0.6196	
MYCB2_2	13	0.8253	0.0005	**0.0080**
SLIT_1	14	-0.1572	0.5914	
SLIT_2	14	0.6548	0.011	**0.0880**
TOP2A_1	14	0.7440	0.0023	**0.0251**
TOP2A_2	14	0.4271	0.1277	

Supplementary Table S1. Spearman rank correlation (rS) between genomic abundance of ten genes and the number of B chromosomes, found in qPCR experiments in gDNA of males with 0-3 B chromosomes. rS= Spearman correlation coefficient, P = P-value, Pb = Sequential Bonferroni-corrected P. Significant effects (Pb < 0.05) are marked in bold-type letter.

Gene	Sex	Item	N	H	df	P	Pb
CIP2A	F	B	18	10.80	3	0.0129	**0.0129**
		G	21	10.82	3	0.0127	**0.0255**
	M	B	23	12.20	3	0.0067	**0.0202**
		G	23	16.36	3	0.0010	**0.0038**
CKAP2_1	F	B	20	2.53	3	0.4698	
		G	21	1.17	3	0.7597	
	M	B	22	1.16	3	0.7624	
		G	22	1.28	3	0.7333	
CKAP2 _2	F	B	21	14.78	3	0.0020	**0.0040**
		G	21	6.66	3	0.0835	**0.0835**
	M	B	23	15.07	3	0.0018	**0.0070**
		G	23	14.94	3	0.0019	**0.0056**
CAP-G_1	F	B	21	11.15	3	0.0110	**0.0110**
		G	21	12.31	3	0.0064	**0.0128**
	M	B	23	17.98	3	0.0004	**0.0018**
		G	23	17.00	3	0.0007	**0.0021**
CAP-G_2	F	B	21	1.91	3	0.5915	
		G	21	1.81	3	0.6124	
	M	B	23	4.63	3	0.2013	
		G	23	1.77	3	0.6218	
GTPB6	F	B	21	1.30	3	0.73021	
		G	21	4.57	3	0.20629	
	M	B	22	1.92	3	0.58933	
		G	23	4.55	3	0.20766	
HYI_1	F	B	21	4.16	3	0.24479	
		G	21	4.41	3	0.22084	
	M	B	21	1.75	3	0.62688	
		G	23	2.27	3	0.51743	
HYI _2	F	B	21	1.23	3	0.74647	
		G	21	5.35	3	0.14764	
	M	B	23	4.21	3	0.23922	
		G	23	4.59	3	0.20412	
KIF20A	F	B	19	6.89	3	0.07551	0.15101
		G	20	1.94	3	0.585	0.585
	M	B	23	13.85	3	0.00311	**0.01244**
		G	23	10.61	3	0.01406	**0.04218**
							**Continue in next page**

Continued from previous page							
**Gene**	**Sex**	**Item**	**N**	**H**	**df**	**P**	**Pb**
MTG1	F	B	21	3.39	3	0.33536	
		G	21	3.70	3	0.29519	
	M	B	23	5.20	3	0.15749	
		G	23	6.17	3	0.10364	
MYCB2_1	F	B	21	4.92	3	0.17784	
		G	20	3.45	3	0.32764	
	M	B	23	0.60	3	0.89539	
		G	23	3.13	3	0.3717	
MYCB2_2	F	B	19	8.73	3	0.03306	**0.03306**
		G	21	10.90	3	0.01228	**0.03685**
	M	B	23	9.84	3	0.01995	**0.03989**
		G	23	12.71	3	0.00531	**0.02125**
SLIT_1	F	B	21	5.65	3	0.12979	
		G	21	3.30	3	0.34737	
	M	B	23	0.99	3	0.80247	
		G	23	6.48	3	0.09035	
SLIT_2	F	B	21	4.57	3	0.20629	
		G	21	1.05	3	0.78868	
	M	B	23	1.38	3	0.71046	
		G	23	3.98	3	0.26326	
TOP2A_1	F	B	21	10.09	3	0.0178	0.0712
		G	21	7.08	3	0.0693	0.1387
	M	B	23	7.08	3	0.0695	0.0695
		G	23	7.71	3	0.0523	0.1569
TOP2A_2	F	B	22	2.14	3	0.5447	
		G	22	0.69	3	0.8750	
	M	B	23	5.13	3	0.1628	
		G	23	4.77	3	0.1892	

Supplementary Table S2. Kruskal-Wallis analysis of qPCR gene expression experiments in males and females with 0-3 B chromosomes. Sex: M = Male, F = Female; Item: B = Body, G = Gonad; N= Number of individuals, df= degrees of freedom, Pb = Sequential Bonferroni P-value

Supplementary figures

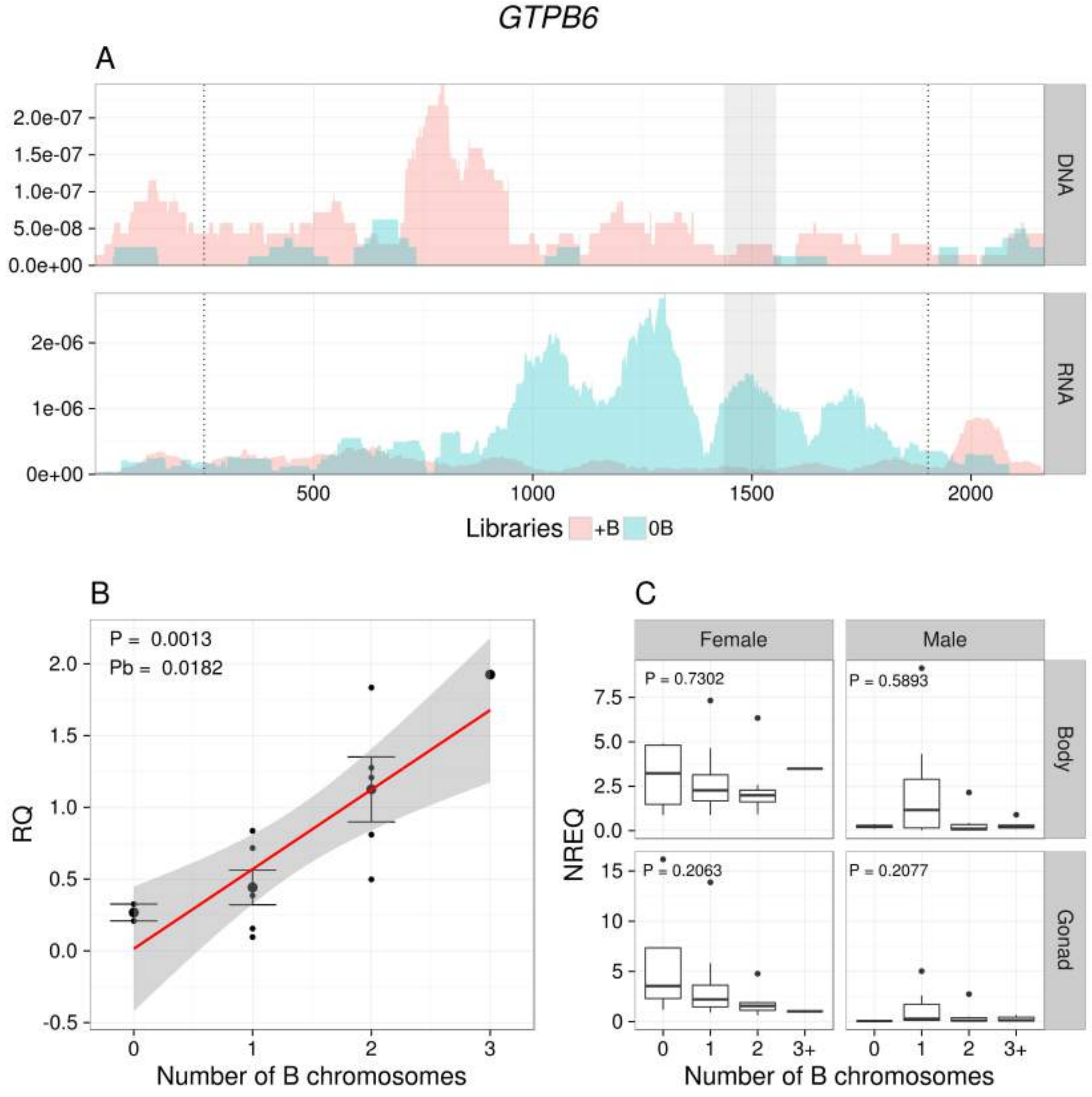

Supplementary Figure S1. Coverage for the *GTPB6* transcript in the gDNA (0B and 4B) and RNA (0B and 1B) Illumina reads (A), and qPCR on gDNA (B) and cDNA (C). Note that coverage was higher in the 4B gDNA library along all sequence length, but it was very low in the B-carrying RNA library (A). The shaded zone in A marks the region amplified by qPCR. qPCR on gDNA (B) revealed that genomic copy number for the *GTPB6* gene increases with B chromosome number following a dose-dependent pattern, thus confirming its presence in the B chromosome. qPCR on cDNA (C) revealed that *GTPB6* expression was independent of B chromosome number in all tissues and sexes analyzed, suggesting that B chromosome gene copies are silenced. RQ = Relative quantity. NREQ = Normalized relative expression quantity. P= P-value and Pb= Sequential Bonferroni P-value for Spearman rank correlation (B) and Kruskal-Wallis (C) analyses.

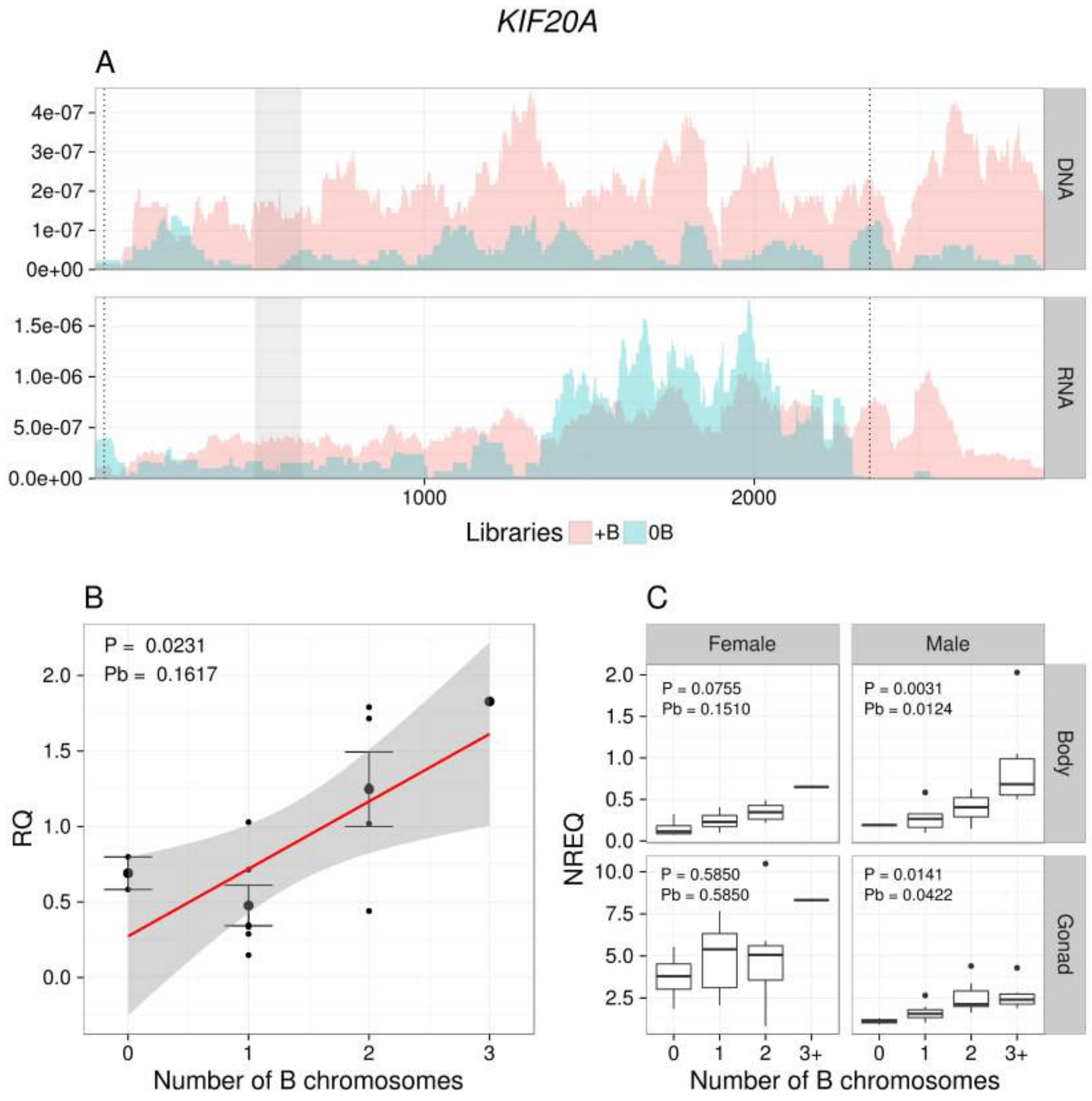

Supplementary Figure S2. Coverage for the *KIF20A* transcript in the gDNA (0B and 4B) and RNA (0B and 1B) Illumina reads (A), and qPCR on gDNA (B) and cDNA (C). Note that coverage was higher in the 4B gDNA library along all sequence length, but not in the B-carrying RNA library (A). The shaded zone in A marks the region amplified by qPCR. qPCR on gDNA (B) revealed that genomic copy number for the *KIF20A* gene increases with B chromosome number following a dose-dependent pattern, thus confirming its presence in the B chromosome, although this association was only marginally significant. qPCR on cDNA (C) showed that *KIF20A* expression tended to increase with B chromosome number only in males. RQ = Relative quantity. NREQ = Normalized relative expression quantity. P = P-value and Pb = Sequential Bonferroni P-value for Spearman rank correlation (B) and Kruskal-Wallis (C) analyses.

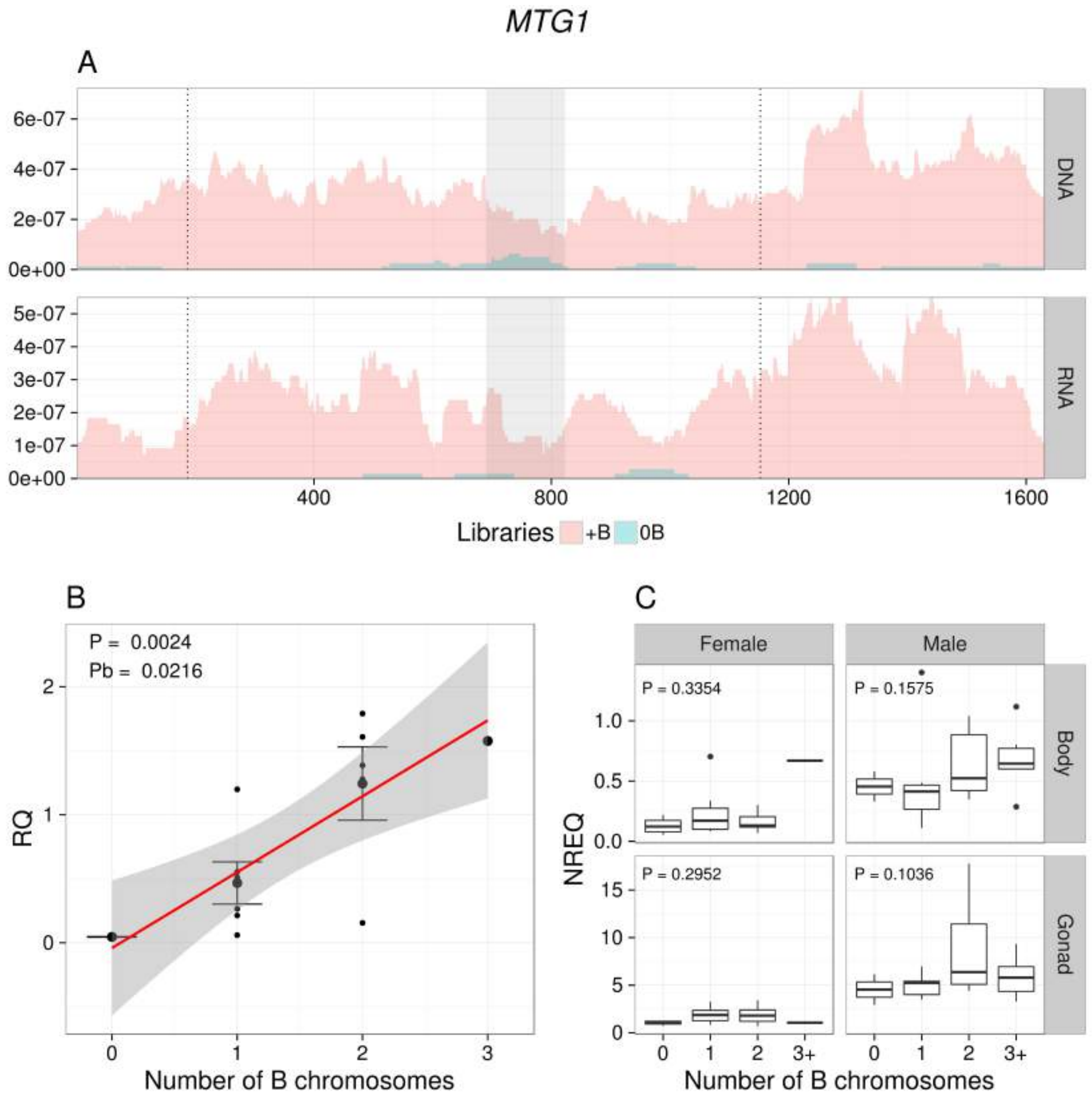

Supplementary Figure S3. Coverage for the *MTG1* transcript in the gDNA (0B and 4B) and RNA (0B and 1B) Illumina reads (A), and qPCR on gDNA (B) and cDNA (C). Note that coverage was higher in the 4B gDNA library along all sequence length, and also in the B-carrying RNA library (A). The shaded zone in A marks the region amplified by qPCR. qPCR on gDNA (B) revealed that genomic copy number for the *MTG1* gene increases with B chromosome number following a dose-dependent pattern, thus confirming its presence in the B chromosome. qPCR on cDNA (C) showed that *MTG1* expression was independent on the number of B chromosomes in all samples, suggesting that this B chromosome gene is silenced. RQ = Relative quantity. NREQ = Normalized relative expression quantity. P= P-value and Pb= Sequential Bonferroni P-value for Spearman rank correlation (B) and Kruskal-Wallis (C) analyses.

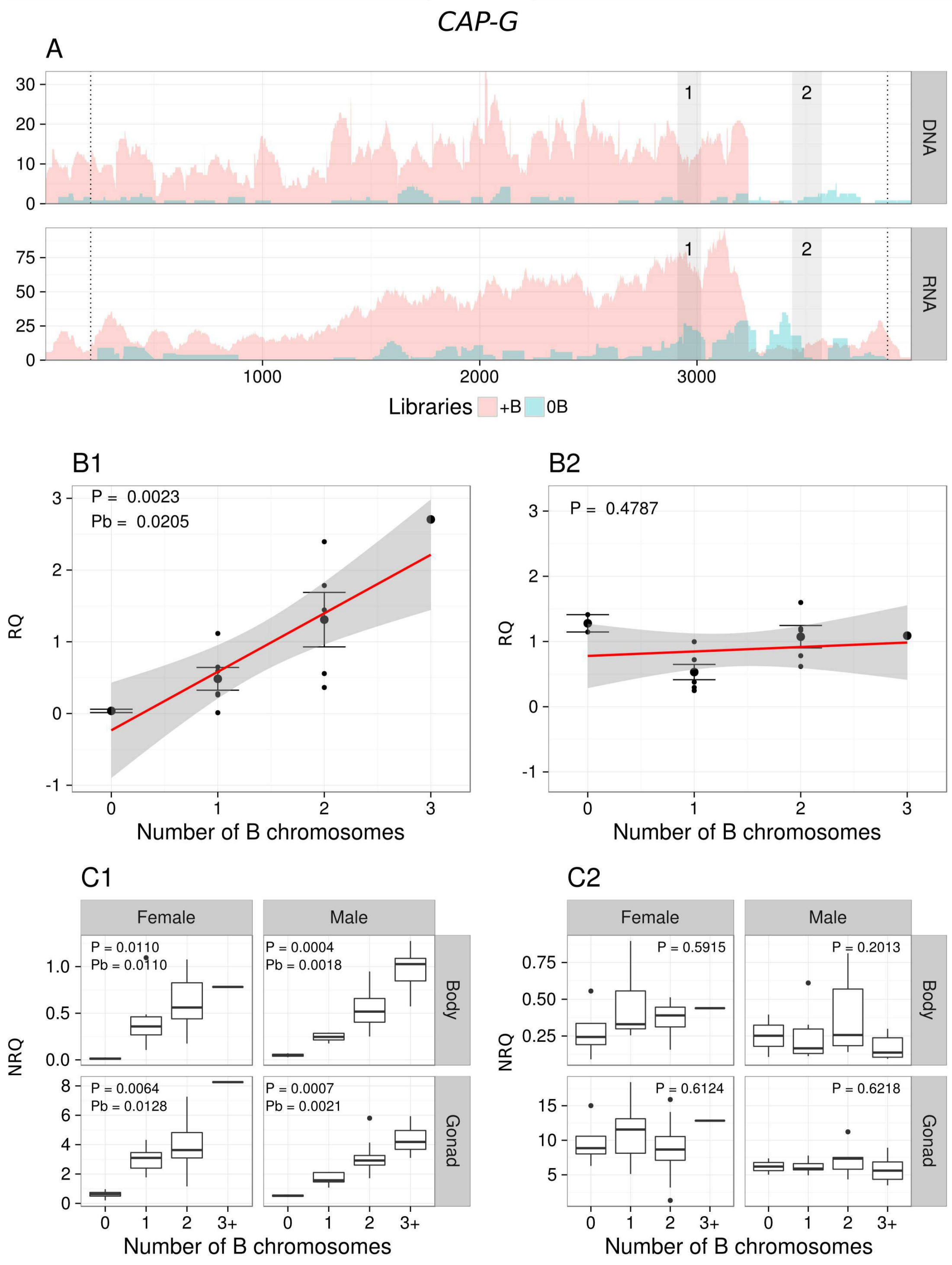

Supplementary Figure S4. Coverage for the *CAP-G* transcript in the gDNA (0B and 4B) and RNA (0B and 1B) Illumina reads (A), and qPCR on gDNA (B) and cDNA (C). Low coverage in the 4B genome for the last five exons (20th-24th) suggests that the CDS of this B chromosome gene (delimited by dotted vertical lines) is truncated. Two regions were selected for qPCR amplification of this gene, one within the region being present in the B chromosome (shaded zone 1) and the other within the region being absent in it (shaded zone 2). qPCR on gDNA with zone 1 primers showed that copy number for this gene region increased with the number of B chromosomes (B1), whereas qPCR

on gDNA with zone 2 primers was independent on B number (B2). Likewise, qPCR on cDNA showed that *CAP-G* expression increased in a dosage-dependent pattern for zone 1 primers (C1) but it was independent of B chromosome number for zone 2 primers (C2), suggesting the active transcription of B chromosome truncated gene copies. RQ = Relative quantity. NREQ = Normalized relative expression quantity. P= P-value and Pb= Sequential Bonferroni P-value for Spearman rank correlation (B) and Kruskal-Wallis (C) analyses.

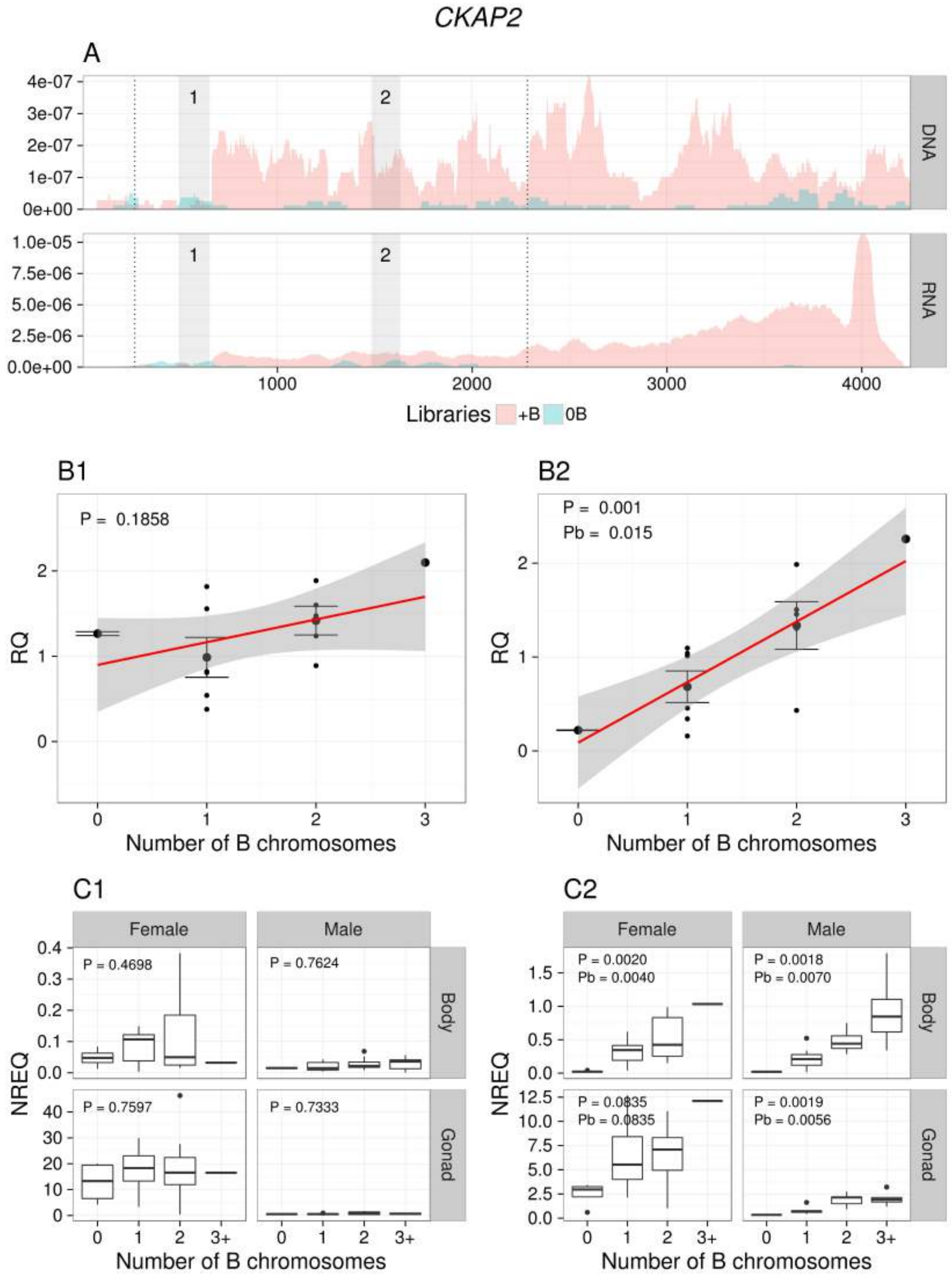

Supplementary Figure S5. Coverage for the *CKAP2* transcript in the gDNA (0B and 4B) and RNA (0B and 1B) Illumina reads (A), and qPCR on gDNA (B) and cDNA (C). Low coverage in the 4B genome for the 5' region of the CDS (till nucleotide 663), suggests that this gene is truncated in the B chromosome. Two regions were selected for qPCR amplification of this gene, one within the region being missing in the B chromosome (shaded zone 1) and the other within the region being present in it (shaded zone 2). qPCR on gDNA with zone 1 primers showed that copy number for this gene was independent on the number of B chromosomes (B1), whereas qPCR on gDNA with zone 2 primers

showed that it increased with the number of B chromosomes (B2), showing that this B chromosome gene is truncated. qPCR on cDNA showed that *CKAP2* expression was independent of B chromosome number for region 1 (C1) but increased in a dosage-dependent pattern for region 2 (C2), suggesting the active transcription of B chromosome truncated gene copies. RQ= Relative quantity. NREQ = Normalized relative expression quantity. P= P-value and Pb= Sequential Bonferroni P-value for Spearman rank correlation (B) and Kruskal-Wallis (C) analyses.

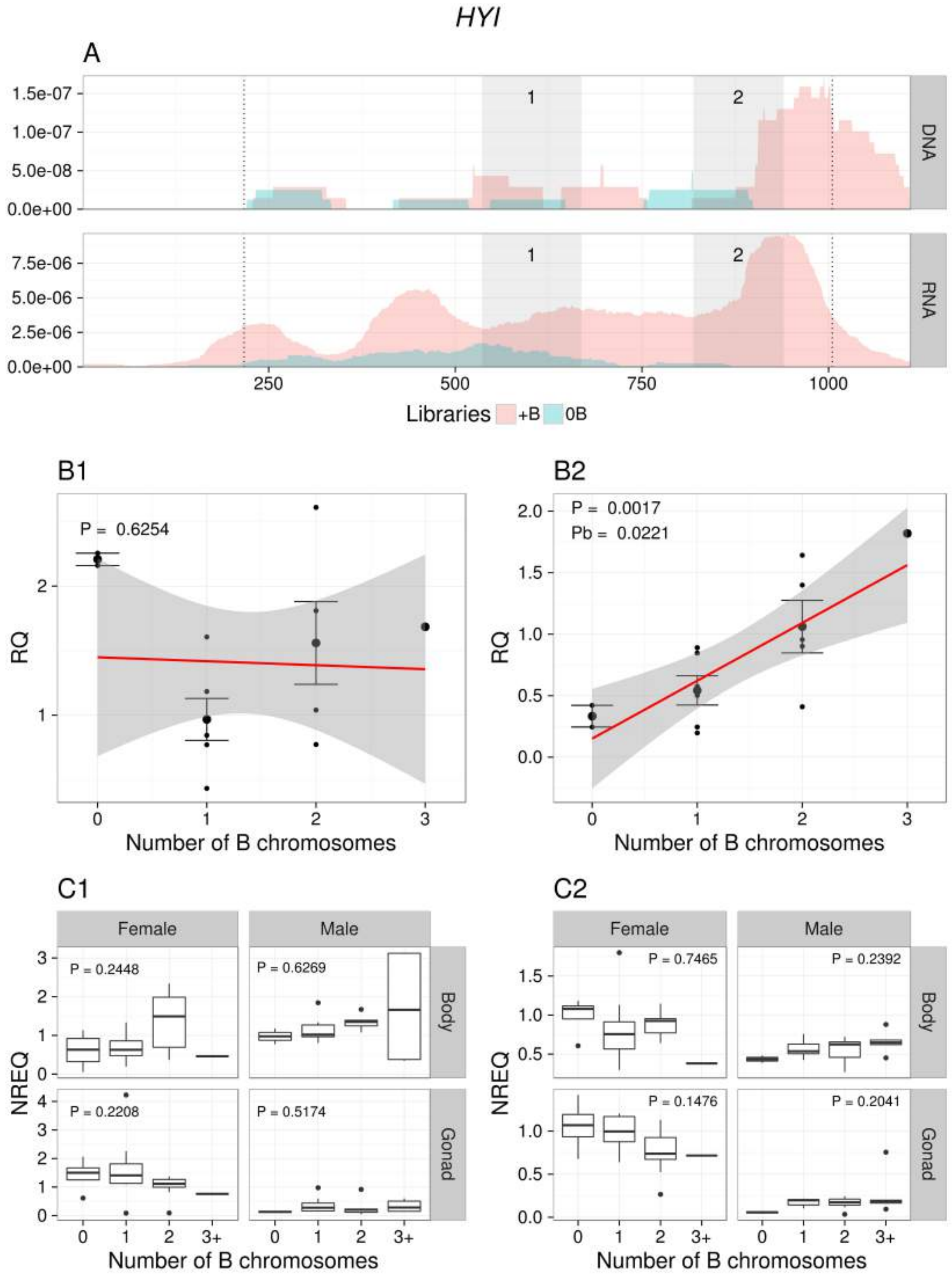

Supplementary Figure S6. Coverage for the *HYI* transcript in the gDNA (0B and 4B) and RNA (0B and 1B) Illumina reads (A), and qPCR on gDNA (B) and cDNA (C). Low coverage in the 4B genome for the 5' region of the CDS (till nucleotide 901) suggests that this gene is truncated in the B chromosome. Two regions were selected for qPCR amplification of this gene, one within the region being missing in the B chromosome (shaded zone 1) and the other within the region being present in it (shaded zone 2). qPCR on gDNA with zone 1 primers showed that copy number for this region was independent on the number of B chromosomes (B1), whereas qPCR on gDNA with zone 2 primers

showed that it increased with the number of B chromosomes (B2), thus showing that this B chromosome gene is truncated. qPCR on cDNA showed that *HYI* expression was independent of B chromosome number for both regions (C1 and C1), suggesting B chromosome truncated copies are inactive. RQ = Relative quantity. NREQ = Normalized relative expression quantity. P= P-value and Pb= Sequential Bonferroni P-value for Spearman rank correlation (B) and Kruskal-Wallis (C) analyses.

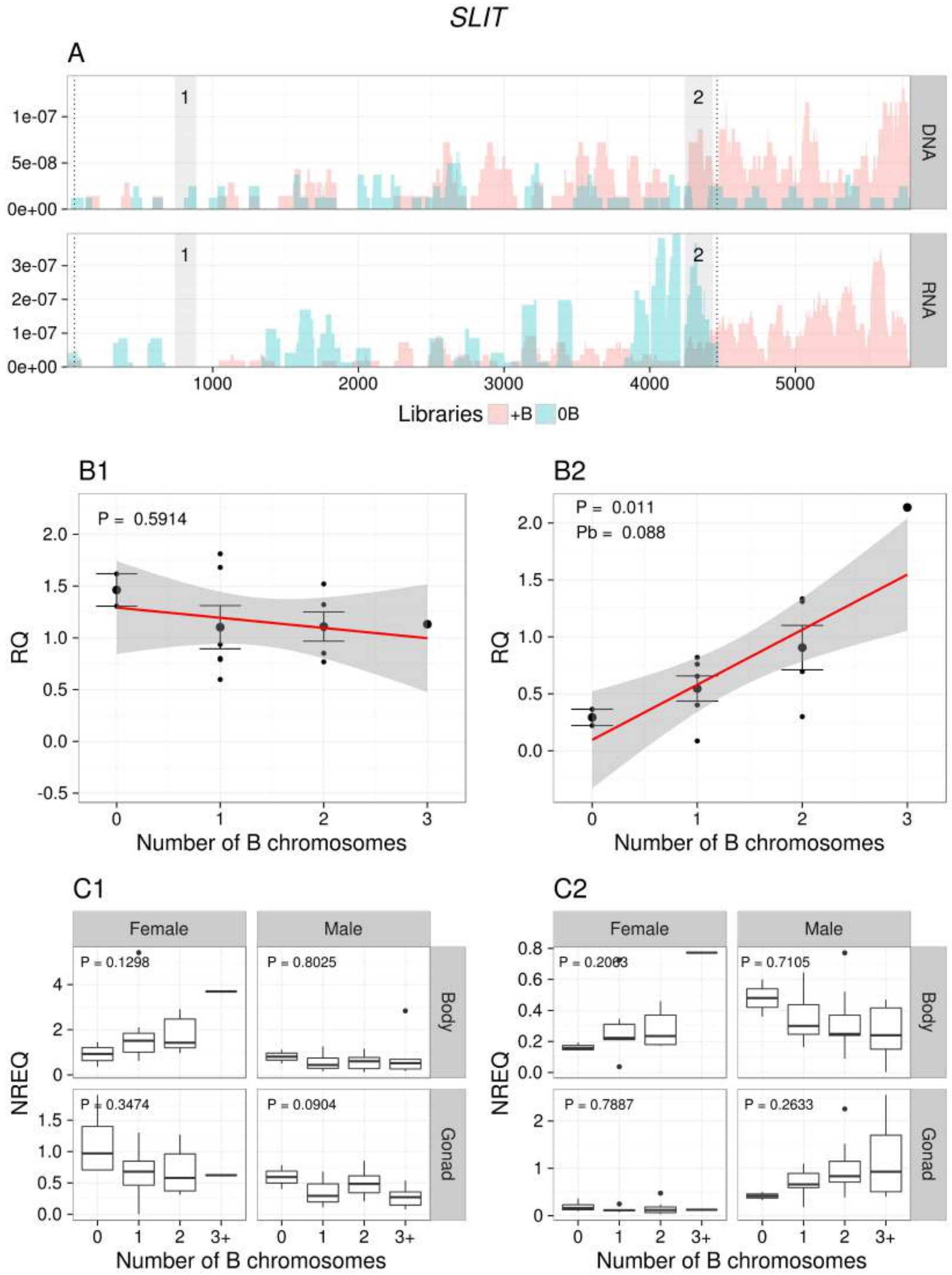

Supplementary Figure S7. Coverage for the *SLIT* transcript in the gDNA (0B and 4B) and RNA (0B and 1B) Illumina reads (A), and qPCR on gDNA (B) and cDNA (C). Coverage in the 4B genome was high for only a small part of the 5' region of the CDS, suggesting that this gene is truncated in the B chromosome (A). Two regions were selected for qPCR amplification of this gene, one within the region being absent in the B chromosome (shaded zone 1) and the other within the region being present in it (shaded zone 2). qPCR on gDNA with zone 1 primers showed that copy number for this gene was independent on the number of B chromosomes (B1), whereas qPCR on gDNA with zone 2 primers showed that it increased with the number of B chromosomes (B2), thus

demonstrating that B chromosome copies are truncated. qPCR on cDNA showed that *SLIT* expression was independent of B chromosome number for both regions (C1 and C1), suggesting the B chromosome truncated copies are inactive. RQ = Relative quantity. NREQ = Normalized relative expression quantity. P= P-value and Pb= Sequential Bonferroni P-value for Spearman rank correlation (B) and Kruskal-Wallis (C) analyses.

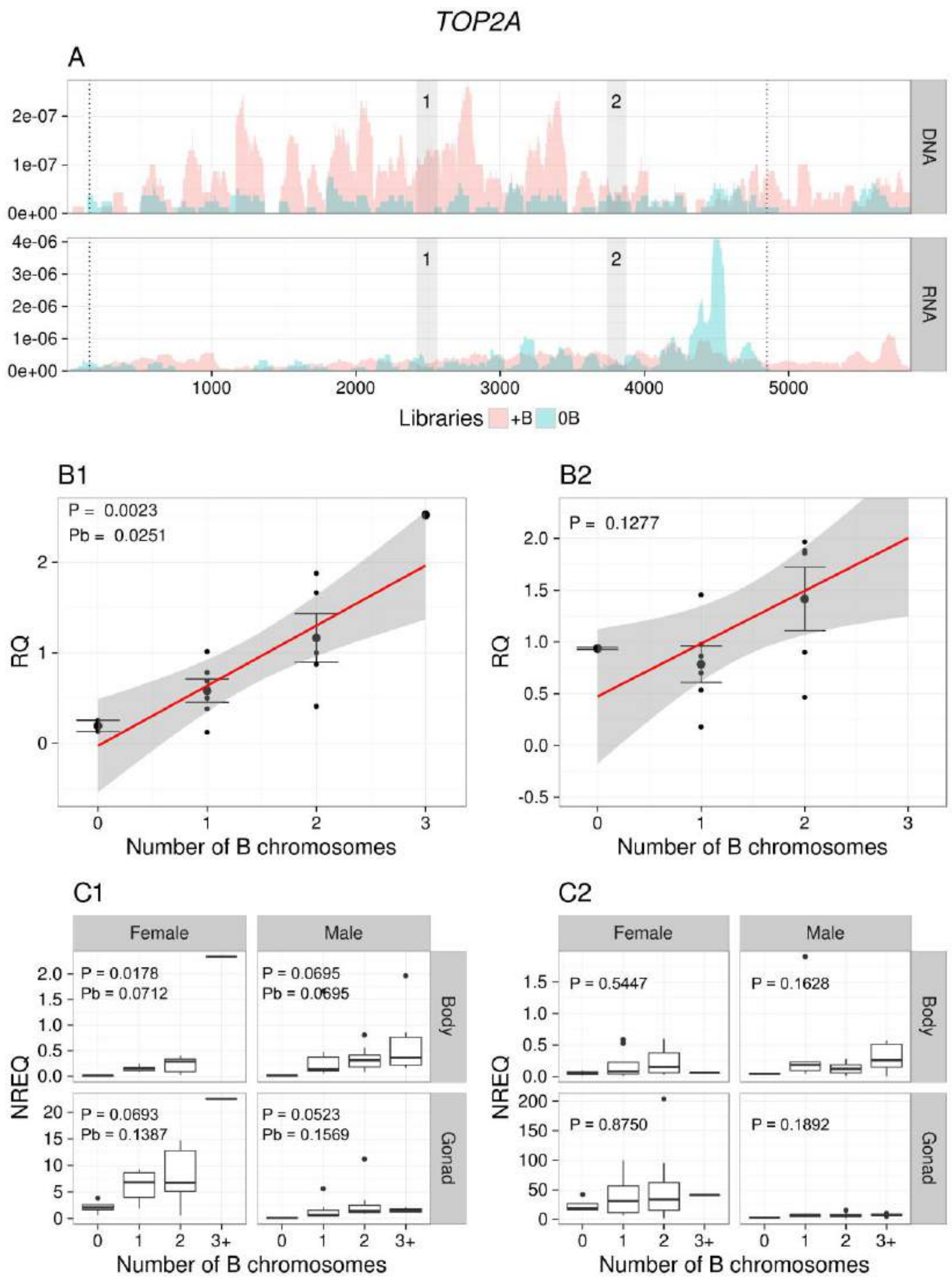

Supplementary Figure S8. Coverage for the *TOP2A* transcript in the gDNA (0B and 4B) and RNA (0B and 1B) Illumina reads (A), and qPCR on gDNA (B) and cDNA (C). Coverage in the 4B genome was high for only a small part of the 5' region of the CDS, suggesting that this gene is truncated in the B chromosome (A). Two regions were selected for qPCR amplification of this gene, one within the region being absent in the B chromosome (shaded zone 1) and the other within the region being present in it (shaded zone 2). qPCR on gDNA with zone 2 primers showed that copy number for this gene was independent on the number of B chromosomes (B2), whereas qPCR on gDNA with zone 1 primers showed that it increased with the number of B chromosomes (B1), thus

demonstrating that B chromosome copies are truncated. qPCR on cDNA showed that *TOP2A* expression was independent of B chromosome number for both regions (C1 and C2), suggesting the B chromosome truncated copies are inactive. RQ = Relative quantity. NREQ = Normalized relative expression quantity. P= P-value and Pb= Sequential Bonferroni P-value for Spearman rank correlation (B) and Kruskal-Wallis (C) analyses.

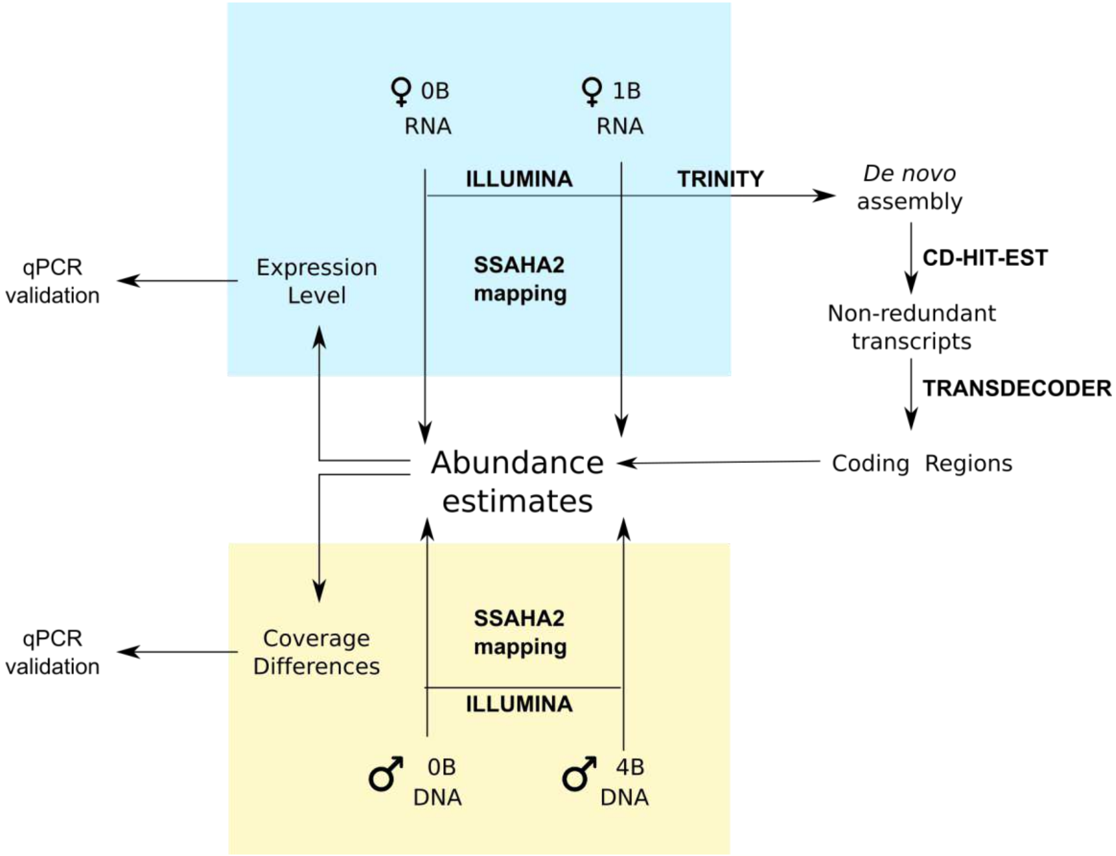

Suplementary Figure S9. Workflow of analysis. Two libraries of llumina reads were obtained by sequencing total RNA from a 0B and a 1B females of *E. plorans*. Reads were pooled and assembled using Trinity, following the guidelines described in Haas *et al*.[57]. After reducing redundancy with CD-HIT-EST[58], we predicted a set of potentially protein-coding regions using Transdecoder[57]. Using the identified protein-coding regions as reference, we mapped against this transcriptome the reads obtained by Illumina sequencing of gDNA extracted from a 0B and a 4B *E. plorans* males, using SSAHA2[62]. We then used a custom script to count the number of mapped reads on each nucleotide position as a measure of abundance. Comparing abundance along the coding region on 0B and 4B gDNA libraries we were able to detect coding regions being over-represented in the 4B library, compared to the 0B one, as an indication of their putative presence in the B chromosome. In addition, we mapped the reads coming from RNA 0B and 1B libraries against the same reference. Comparison of mapped read abundance between RNA 0B and 1B libraries revealed that some of those genes located in the B chromosome were possibly transcribed. Both conclusions (localization of protein-coding genes on the B chromosome and their transcription) were validated by means of qPCR.
